# L2 Learners Do Not Ignore Verb’s Subcategorization Information in Real-Time Syntactic Processing

**DOI:** 10.3389/fpsyg.2021.689137

**Published:** 2022-01-20

**Authors:** Chie Nakamura, Manabu Arai, Yuki Hirose, Suzanne Flynn

**Affiliations:** ^1^Global Center for Science and Engineering, Waseda University, Tokyo, Japan; ^2^Faculty of Economics, Seijo University, Tokyo, Japan; ^3^The Graduate School of Arts and Sciences, The University of Tokyo, Tokyo, Japan; ^4^Department of Linguistics, Massachusetts Institute of Technology, Cambridge, MA, United States

**Keywords:** eye-tracking in reading, filler-gap dependency, verb subcategorization information, online structural analysis, second language processing

## Abstract

This study addressed the question of whether L2 learners are able to utilize verb’s argument structure information in online structural analysis. Previous L2 research has shown that L2 learners have difficulty in using verb’s intransitive information to guide online syntactic processing. This is true even though L2 learners have grammatical knowledge that is correct and similar to that of native speakers. In the present study, we contrasted three hypotheses, the initial inaccessibility account, the intransitivity overriding account, and the fuzzy subcategorization frame account, to investigate whether L2 learner’s knowledge of intransitive verbs is in fact ignored in L2 online structural analysis. The initial inaccessibility account and the fuzzy subcategorization frame account predicted that L2 learners cannot access intransitivity information in building syntactic structures in any situation. The intransitivity overriding account predicted that intransitivity information is accessed in L2 parsing, but this process is overridden by the strong transitivity preference when a verb is followed by a noun phrase. Importantly, the intransitivity overriding account specifically predicted that L2 learners would be able to use intransitive information in online syntactic processing when a noun phrase does not appear immediately following a verb. We tested the three accounts in an eye-tracking reading experiment using filler-gap dependency structures. We manipulated verb’s transitivity information and lexically based plausibility information and tested English native speakers as a control L1 group (*N* = 29) and Japanese-English L2 participants (*N* = 32). The results showed that L2 learners as well as native speakers processed sentences differently depending on the subcategorization information of the verb, and adopted transitive analysis only when the verb was optionally transitive, providing support for the intransitivity overriding. The results further demonstrated that L2 learners had strong expectations for the transitive structure, which is consistent with the view proposed by the hyper-active gap-filling hypothesis. In addition, the results showed that the semantic mismatch in the incorrect transitive analysis facilitated native speaker’s processing but caused difficulty for L2 learners. Together, the current study provides evidence that L2 learners use intransitive information of the verbs to guide their structural analysis when there are no overriding constraints.

## Introduction

Individual verbs contain information about which structure they can appear in. For example, the verb *listen* possesses information that it can occur in the intransitive structure but cannot occur in the transitive structure while the verb *hear* is the other way around. This is called a verb’s argument structure or subcategorization frame information. It is often assumed that language users use this information during real-time language comprehension to analyze a sentence structure. It is, however, still not clear whether this holds for second language (L2) learners. Specifically, it is still under debate whether L2 learners possess the same lexically specific knowledge as that of native speakers. This is an important question as it relates to larger questions such as whether there is a qualitative difference between L1 and L2 processing, and to what extent native speakers and L2 learners share the same processing mechanisms beyond the difference in their general language proficiency. The current study addressed these questions by testing the effect of verb subcategorization information in the process of syntactic ambiguity resolution with native English speakers and Japanese speakers learning English.

In first language (L1) processing, several studies have shown that verb’s structural frequency information is used in structural building operations, providing evidence that subcategorization frames and frequency information associated with each verb are used at the very early stage in sentence processing (MacDonald et al., 1994; McRae et al., 1998 among others). For example, Trueswell et al. (1993) used sentences such as example in (1) and compared verbs that typically take a direct object (1a, *forget*) to verbs that rarely take a direct object (1b, *hope*).

(1a) The student forgot the solution was in the back of the book.
(1b) The student hoped the solution was in the back of the book.

In a self-paced reading experiment, they observed longer reading times at the region following the point of the disambiguation (i.e., *in*) in (1a) than in (1b), demonstrating that comprehenders committed more strongly to the incorrect direct object analysis in (1a) than in (1b). These results, along with those from other studies (e.g., Trueswell, 1996; Garnsey et al., 1997), suggest that native speakers use verb bias information to resolve structural ambiguities during online comprehension.

However, there are also studies that failed to observe an immediate effect of verb information in online structural analyses with native speakers. While these studies also assume a major role for verb information, they argue that verb information does not guide the initial parsing operation. For example, Pickering et al. (2000) tested sentences such as in example (2), in which the verb *realize* was biased toward the sentence complement. In eye-tracking experiments, they observed longer reading times at the post-verbal noun phrase (NP) in (2b, *her exercises*) than in (2a, *her potential*).

(2a) The young athlete realized her potential 1 day might make her a world-class sprinter.
(2b) The young athlete realized her exercises 1 day might make her a world-class sprinter.

The results showed that comprehenders initially analyzed the post-verbal NP as the verb’s direct object even though the verb’s structural frequency information was biased against the analysis and they experienced processing difficulty when the interpretation for the direct object analysis was semantically implausible. This suggests that comprehenders did not consider verb frequency information at the initial stage of processing and adopted a direct object analysis (see also Mitchell, 1987; Ferreira and Henderson, 1990; Kennison, 2001; Pickering and Traxler, 2003; for similar results).

These studies appear to be at odds with the other studies which observed an immediate effect of the verb’s structural information in online comprehension. However, there is one possible interpretation that can reconcile the two different patterns of results; the results that failed to show an immediate effect of verb information do not necessarily mean that comprehenders ignore verb information in early processing. It is possible that although lexical information is accessed immediately upon encountering the verb, it is overridden by the preference for the most frequent direct object analysis when an NP directly follows the verb. In fact, this possibility has been supported by Arai and Keller (2012), who investigated eye movements reflecting predictive structural analysis in sentence processing. In a visual world eye-tracking paradigm, they manipulated verb types with different subcategorization frames such as in example (3) and showed that on encountering the verb (e.g., *punished*/*disagreed*) participants immediately looked more at an object that can serve as the verb’s direct object in the visual scene (e.g., artist) in (3a) than in (3b).

(3a) Surprisingly, the nun punished the artist.
(3b) Surprisingly, the nun disagreed with the artist.

Their results provide evidence that comprehenders made different predictions based on the verb’s subcategorization information. In the same study, they also tested whether frequency information that a particular verb that is used more frequently in a past participle form or in a main verb form plays a role in structural prediction. The results showed that participants predicted the correct sentence structure based on the verb’s frequency information. These results support the view that the verb’s lexically specific information about subcategorization information and frequency information, as well as the distribution of morphological forms are immediately accessed at the earliest stages of processing during online comprehension.

In L2 processing, the evidence for the use of the verb’s structural information is relatively scarce. For example, Dussias and Cramer Scaltz (2007) examined whether Spanish-English L2 learners used verb bias information in processing sentences such as in example (4). The verb was either biased toward a direct object as in (4a), or toward a sentence complement as in (4b).

(4a) The CIA director confirmed the rumor could mean a security leak.
(4b) The ticket agent admitted the mistake when he got caught.

Their results from their self-paced reading experiments showed that L2 learners experienced processing difficulty when the verb was followed by a constituent that was inconsistent with the verb’s structural bias. They also found that for the verbs whose bias differed between Spanish and English, L2 learners processed them based on their L1 verb bias. (For similar results with Chinese-English L2 learners, see Juffs and Harrington, 1995; Juffs and Harrington, 1996, with French-English L2 learners, see Frenck-Mestre and Pynte, 1997). These results suggest that L2 learners can access verbal information but it remains unclear whether the information L2 learners accessed in processing L2 sentences was the lexical information of the L1 or that of the L2 (but see Lee et al., 2013 for the finding of an effect of the verb’s structural frequency information with advanced Korean- English L2 learners).

Some studies are clearly inconsistent with the view that L2 learners access verb subcategorization information in sentence processing. Nakamura et al. (2013), for example, tested Japanese-English L2 participants in processing temporarily ambiguous sentences such as example (5), in which the verb was either optionally transitive (5a, *watch*) or obligatory intransitive (5b, *cry*).

(5a) When the audience watched the actor rested behind the curtain.
(5b) When the audience cried the actor rested behind the curtain.

Their results from self-paced reading studies showed that the L2 learners initially analyzed a post-verbal NP as the verb’s direct object both in (5a) and (5b), demonstrating that L2 learners ignored the verb’s subcategorization information, viz. the information that the verb *cry* cannot take a direct object. L2 learners always adopted a direct object analysis initially regardless of whether the analysis was licensed by the verb’s subcategorization information or not.

Using the same early/late closure ambiguity, Nakamura et al. (2019) observed that L2 learners adopted the direct object analysis both with (5a) and (5b), replicating their earlier study. Furthermore, they found that patterns of a priming effect were different for (5a) and (5b). After reading (5a), the processing cost in reanalysis was reduced in reading the subsequent target sentence that had the same verb. No such learning effect was observed with (5b), in which the verb was obligatory intransitive. Their findings suggest that the reading patterns in the prime sentences were ostensibly the same between (5a) and (5b) but the structure the L2 learners activated in reading these sentences was different depending on the verb’s subcategorization information, which influenced the processing of the subsequent target sentences. Importantly, they also confirmed in an off-line task that their L2 learners possessed the correct knowledge regarding subcategorization frames for the verbs used in their study.

In summary, although the results of previous studies that tested L2 learners’ use of verb information might be attributed to various factors such as similarities between the learners’ L1 and L2, learner’s proficiency level, and learner’s cognitive capacity limitations (e.g., Dekydtspotter et al., 2006; Hopp, 2010), past L2 studies largely agree that L2 learners cannot use verbal information as reliably as native speakers do and this holds true even with L2 learners at an advanced level of competence (e.g., Hoover and Dwivedi, 1998; Jiang, 2004, 2007) and regardless of the similarities between the learner’s L1 and L2 (e.g., Papadopoulou and Clahsen, 2003; Marinis et al., 2005; Roberts and Felser, 2011).

The verbal structural frequency information, such that *accused* is frequently used as a participle form but *searched* is hardly ever used as a participle form, is based on statistical distributions that are learned through linguistic input (Francis and Kucera, 1982). It is, therefore, reasonable to think that the verb’s structural frequency information in an L2 is difficult to master perfectly because the majority of L2 learners are exposed to far less linguistic input in the target language compared to native speakers. This might account for the results of some of the research that found similar effects in the use of verb frequency information between native English speakers and advanced L2 learners who were living in an English-speaking country at the time of testing (Lee et al., 2013). However, it does not explain why L2 learners also show difficulty in using syntactic restriction information about which structure a particular verb can appear in, viz. the information that intransitive verbs cannot take a direct object (*intransitivity information* henceforth), in online processing even though they have the correct subcategorization knowledge in the L2. To be more specific, the studies by Nakamura et al. (2013, 2019) suggest that L2 learners do possess the correct subcategorization frame information for obligatory intransitive verbs but it seems problematic for them to apply the knowledge in online structural analyses; they adopted the transitive analysis with the obligatory intransitive verbs. One possible explanation for this outcome comes from previous L1 research in English which suggests that intransitivity information is distributional information that is associated with a specific verb, and this information can be learned only through linguistic experience. In Van Gompel et al. (2012), they demonstrated that the information about whether a specific verb should be used in an intransitive structure or not is represented at a lexically specific level, whereas transitivity information is the default, category-general information (see also Van Gompel et al., 2006). This predicts that L2 learners of English are strongly influenced by the general transitive bias of the verbs and the impact of verb-specific intransitive bias remains small due to the overall shortfall of linguistic input.

One piece of evidence for L2 learners’ strong preference for the transitive structure comes from work by Roberts and Felser (2011). They tested sentences such as example (6) with advanced Greek-English L2 learners.

(6a) While the band played the song pleased all the customers.
(6b) While the band played the beer pleased all the customers.

The results showed that L2 learners analyzed the post-verbal NP as the verb’s direct object and experienced large processing difficulty when the direct object interpretation was semantically implausible in (6b, *played the beer*), unlike the control native speaker group who were able to quickly revise the sentence structure for the correct analysis in which the NP (the beer) is a main clause subject, when the initially adopted direct object interpretation was semantically implausible. This processing pattern most likely reflects the fact that L2 learners relied more on semantic information than on the verb’s subcategorization information. Since L2 learners’ knowledge about intransitive use of optionally transitive verbs such as *play* was weak or unreliable, they were not able to abandon the semantically implausible direct object analysis. This caused L2 learners’ reanalysis process to be delayed or blocked by semantic information (see also Juffs and Harrington, 1996; Juffs, 1998; Dekydtspotter and Seo, 2017; for studies that tested the effect of intransitivity information in L2 processing). Their results suggest that L2 learners’ syntactic processing ability, especially the ability to use information that a specific verb can appear in an intransitive structure, is reduced compared to native speakers.

The results of Roberts and Felser (2011), along with other studies that failed to observe a reliable effect of verb subcategorization information in L2 processing, support the view that L2 learners have a strong tendency to analyze an NP that immediately follows a verb as the verb’s direct object. As a consequence, L2 learners tend to create a VP even when the verb’s subcategorization information does not allow the analysis (Juffs and Harrington, 1996; Juffs, 1998; Nakamura et al., 2013, 2019). Again, it is important to note that L2 learner’s grammaticality judgments were similar to the native speaker’s judgments, suggesting that L2 learners had the correct knowledge about the subcategorization bias of the verbs in an off-line task (Juffs, 1998; Nakamura et al., 2019). If L2 learners have similar subcategorization knowledge to that of native speakers, why is this knowledge not reflected in their online processing?

One possible and probably the most straightforward interpretation would be that L2 learners possess the knowledge that particular verbs cannot take a direct object, but L2 learners have difficulty with the immediate use of this information in online processing. Findings from some previous studies suggest that even native speakers initially adopt the transitive analysis on encountering an intransitive verb and experience processing difficulty (cf. Mitchell, 1987). It is possible that the preference for the transitive analysis is even stronger for L2 learners and consequently, with an intransitive verb, L2 learners always initially attempt to adopt the transitive analysis before they can use intransitivity information and experience processing cost. We call this the *initial inaccessibility account*.

The second possibility is that intransitivity information is accessed in L2 learner’s online structural analysis, but intransitivity information is overridden by a strong transitivity preference when L2 learners see an NP following the verb. More specifically, L2 learners can access subcategorization knowledge for intransitive verbs, but the presence of an NP directly following a verb overrides the intransitivity information so that L2 learners adopt the transitive analysis over the intransitive analysis. We call this the *intransitivity overriding account*. In this case, L2 learners are predicted to use intransitivity information in an online structural analysis where an NP does not appear directly following a verb.

The third possibility is that L2 learners’ lexical representation of argument structure information is fuzzy in the sense that L2 information about a certain subcategorization frame is not stored rigidly as either possible or not. Instead, the lexical representation of L2 learners may allow some ambiguity in their structural specifications. As a result, L2 learners may tolerate an incorrect subcategorization frame (e.g., an obligatory intransitive verb to take a direct object) to a greater extent compared to native speakers. Due to the fuzzy structural representations, L2 learners permit subcategorization violation and make semantic interpretations out of the information they receive. We call this the *fuzzy subcategorization frame account*. Under this account it is predicted that L2 learners cannot use verb’s subcategorization information to sort out which structures are possible or not due to the L2 learner’s fuzzy structural representations. Instead, they would rely on the semantic relationship between the verb and an NP. This account is consistent with the view suggested in some previous L2 studies that L2 processing is strongly influenced by lexical-semantic cues but less so by syntactic information (cf. Clahsen and Felser, 2006).

In order to test these accounts, we examined L2 learner’s processing using the unbounded dependency structure such as (7a). In processing this structure, a parser needs to associate the object NP (*the celebrity*), which is referred to as the *filler*, to the correct post-verbal thematic position, called the *gap*. Since the verb *interview* in (7a) is optionally transitive, readers would typically posit an incorrect gap immediately following the verb (i.e., *That’s the celebrity that the writers interviewed___* [*about*.]), by analyzing the NP as a direct object of the verb (i.e., *The writer interviewed the celebrity*). However, this interpretation turns out to be inconsistent with the sentence continuation at the information *at the conference*, and readers are thus forced to reanalyze the structure for the correct intransitive interpretation (i.e., the writer did not interview the celebrity, but she/he interviewed about the celebrity.).

(7a) That’s the celebrity that the writer interviewed about at the conference.
(7b) That’s the letter that the writer interviewed about at the conference.
(7c) That’s the letter that the writer smiled about at the conference.

Using this structure, we investigated the influence of verb-specific information on L2 learner’s initial parsing processes by manipulating transitivity information of the verb in (7). The verb was either optionally transitive (7a, 7b; *interview*) or intransitive (7c; *smile*). In addition, we also manipulated semantic information for the incorrect direct object analysis in two optionally transitive verb conditions (7a, b). In L1 studies, it has been shown that semantically anomalous interpretation helps L1 speakers to quickly abandon the favored analysis and adopt the correct analysis before disambiguation (Pickering and Traxler, 1998). In contrast, there is evidence that L2 learners cannot move beyond the favored analysis even when the interpretation for the analysis is semantically anomalous (Roberts and Felser, 2011). In order to examine whether semantic information helps L2 learners to recover from the incorrect analysis in processing filler-gap dependency structures, we used different nouns for the filler NP. The incorrect gap-filling direct object analysis resulted in either semantically plausible (7a; *interviewed the celebrity*, Plausible transitive condition), or impossible (7b; *interviewed the letter*, Implausible transitive condition).

If the verb’s subcategorization information does not have an influence on L2 processing, the preceding NP (*the celebrity*/*the letter*) would be always analyzed as the verb’s direct object in all conditions. Importantly, the initial inaccessibility account and the fuzzy subcategorization frame account both assume that subcategorization information cannot be used during L2 structural analysis, but these two accounts predict different reading patterns. Under the initial inaccessibility account, L2 learners are predicted to initially attempt to analyze the preceding NP (*the celebrity*/*the letter*) as the verb’s direct object regardless of the verb type. As a result, L2 learners would experience processing difficulty after they encounter the verb both in (7b) and (7c) compared to (7a). In (7b), processing cost would be observed due to the semantically implausible interpretation (*interviewed the letter*). In (7c), L2 learners initially adopt a direct object analysis and experience processing difficulty because the analysis violates the verb’s subcategorization information (*smiled the letter*). Under the fuzzy subcategorization frame account, it is predicted that L2 learners are incapable of making structural judgment based on verb’s intransitivity information, thus they cannot reject the sentence when an intransitive verb is used in a transitive structure. This suggests that L2 learners form an interpretation of the ungrammatical sentence using semantic information, such that they would interpret “smiled the letter” in (7c) meaning something like “smiled about the letter” or “smiled at the letter”. If this were the case, L2 learners would show processing difficulty only in (7b) because the direct object analysis with the optionally transitive verb *interview* generates semantically anomalous interpretation. There would be no processing difficulty in (7c) because L2 learners would tolerate the incorrect transitive use with the intransitive verb and build semantically plausible interpretation (e.g., *the writer smiled at/about the letter*).

In contrast to the two accounts, the intransitivity overriding account predicts that subcategorization information is accessed and used in L2 processing as long as an NP does not appear immediately following a verb. Under this account, the preceding NP would be analyzed as the verb’s potential direct object only when the verb is optionally transitive in (7a, 7b), but not when the verb is intransitive in (7c). Thus, this account predicts that processing difficulty at the verb would occur only in (7b) due to the semantically implausible direct object analysis but not in (7a) and (7c).

Using these sentences, we conducted an eye-tracking experiment with native speaker of English and Japanese L2 learners of English. In what follows, we will first describe how the experiments were conducted in “Materials and Methods” section. We will then explain how the analyses were conducted and report the results.

## Materials and Methods

### Participants

Twenty-nine native speakers of English (L1 group) and 31 Japanese learners of English (L2 group) participated in the study. Participants of the L1 group were recruited in the Boston area and received a small remuneration for their voluntary participation. All of the participants had normal or corrected-to-normal vision. Participants of the L2 group were adult Japanese-L1 English-L2 speakers living in Japan. They were all undergraduate students at the University of Tokyo, who had at least 6 years of English education in junior high and high school before enrolling in the university. We obtained L2 participants’ scores for the standardized English test in the National Center Test for University Admissions (*mean score* = 194.8 out of 200, *SD* = 7.30). Our L2 participants’ scores corresponded to the proficiency level of B2 to C1 (Independent user level to Proficient user level) on the Common European Framework of Reference for Languages (CEFR) (Council of Europe [COE], 2021).

### Materials and Design

We created 24 sets of experimental items in three conditions (Plausible transitive, Impossible transitive, and Intransitive) as shown again below in example (7). Regions were divided as indicated by the region numbers. These regions were used for the purpose of analysis and were not presented in the experiment. The complete set of target items used in the experiment is shown in Supplementary Table 1.


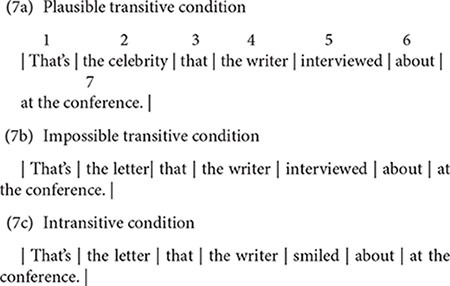


### Procedure

Three lists of items were created following a Latin square design. Each list included 48 fillers and was presented in a pseudo-random order. The filler sentences were structurally unrelated copular sentences. The eye-movements during reading were recorded using EyeLink 1000 (SR Research) for the L1 group, and Eye-Link II (SR Research) for the L2 group. In both experimental settings, a 21” LCD monitoring screen was placed approximately 55 cm away from participants and participants’ eye-movements were recorded at the sampling rate of 500 Hz. A brief calibration set-up was conducted at the beginning of each experimental session. Before each trial, participants saw a square box in the position of the first character of a sentence, which triggered the presentation of sentences. They pressed the space bar when they had finished reading the whole sentence. Eighteen comprehension questions were included following filler sentences to keep participants focused. None of the questions concerned the understanding of the filler-gap dependency structure. The experiment session always started with four practice sentences along with two comprehension questions.

## Data Analysis and Results

### Methods of Analysis and Results

Prior to the analysis of eye-tracking data, we checked the participants’ response accuracy rate for the comprehension questions. The average correct response rate was 96.8% (*SD* = 17.4) for the L1 group, and 92.6% (*SD* = 2.7) for the L2 group. None of the participants were excluded from the analysis. The eye-tracking data were analyzed in three eye-movement measures; *first-pass*, *right-bound*, and *second-pass* reading times. First pass time is the sum of durations of the fixations in a particular region following the first entry in the region until the first fixation outside the region (either to the left or the right). Right-bounded reading time is the sum of fixation durations in a particular region before the first fixation exiting the region to the right. Second-pass reading time is the sum of fixations made in a region after the region has already been exited to the right. These measures were selected to analyze the initial and second stages of processing. First pass and right-bound times in a given region reflect reading patterns before seeing any information following the region of interest, thus they are considered early measures in the sense that the behavior does not reflect the uptake of information in the following regions. Second pass times reflect reading patterns following the encounter of the region for the second time or later (i.e., re-reading after readers proceeded to the following regions), thus considered as a measure that reflects a later stage of processing such as structural reanalysis. The mean reading times for the three eye-movement measures from Region 2 to Region 7 in each condition for the two groups are shown in Supplementary Table 2.

For statistical analysis, we analyzed the reading times in the three measures in each region using Linear Mixed-Effects (LME) models (Baayen, 2008). In the model, Condition (*Plausible transitive, Impossible transitive, or Intransitive*), Group (L1 or L2), and interaction between the two were included as fixed effects. Participants and items were included as random effects. In Region 2 and Region 5 where different words were used across conditions, the number of characters (Word Length) and word frequency (Frequency) were included in the model as additional control factors. The frequency for the lexical items used in Region 2 and Region 5 was counted using the written part of the British National Corpus (data obtained from http://english-corpora.org/bnc). For the lexical items used in the verb region (Region 5), we only counted instances in which the word was used as a verb. The mean frequency counts for the words used in Region 2 in the Plausible transitive condition and those used in the Impossible transitive/Intransitive conditions were 6,313 (*SD* = 7,034) and 13,748 (*SD* = 12,958) respectively. The mean frequency counts for the verbs used in Region 5 in the Plausible transitive/Impossible transitive conditions and those used in the Intransitive condition were 6,587 (*SD* = 7,026) and 2,937 (*SD* = 2,473) respectively.^1^

We analyzed the three conditions using LME models with dummy coding by treating the Impossible transitive condition as a baseline against which the effects of the other two conditions were tested. The Impossible transitive condition was used as a baseline so that we can examine the effect of semantic plausibility by a comparison between the Impossible transitive condition and the Plausible condition [e.g., *interviewed the letter* vs. *interviewed the celebrity* in example (7)], as well as the effect of verb’s subcategorization information by the comparison between the Impossible transitive condition and the Intransitive condition [e.g., *interviewed the letter* vs. *complained the letter* in example (7)]. In the report, a main effect of Plausible transitive condition reflects an effect of semantically plausible/impossible transitive analysis, and a main effect of Intransitive condition reflects an effect of verb’s subcategorization information. Importantly, an interaction between Group and the two experimental conditions reflects the difference in the use of semantic plausibility information and that of verb’s subcategorization information. The factor Group was also dummy coded in the model in which the L1 group was treated as a baseline. For results that showed an interaction between Group and experimental conditions, we conducted a simple effect analysis using the same model by dummy coding the L2 group as a baseline to explore the significance of the main effect with the L2 group. The initial model included a random slope of the fixed effect for both participant and item random effects. The best-fitting model was explored using a backward selection approach. We excluded data that exceeded two standard deviations above the absolute value of residuals from the best-fitting model (Baayen, 2008).

Table 1 shows the results of the analysis in each region in each measure. *P*-values were obtained using the R package lmerTest, which estimate the degree of freedom *via* the Satterthwaite approximation. Below, we discuss the results in each region.

**TABLE 1:** Results of linear mixed-effects models of the three reading time measures in each region.

	β	*SE*	*t*	*p*
**Region 2 (*the celebrity/the letter*)**				
*• First pass reading time*				
*Intercept* (Baseline: L1group)	212.88	31.02		
Plausible transitive condition	–20.71	35.01	–0.59	0.554
Intransitive condition	–3.35	33.09	–0.10	0.920
**Group**	**340.35**	**36.27**	**9.38**	**<0.001**
Group × Plausible transitive condition	15.96	39.85	0.40	0.689
Group × Intransitive condition	5.48	38.56	0.14	0.887
*• Right–bounded reading time*				
*Intercept* (Baseline: L1 group)	482.49	40.75		
Plausible transitive condition	–44.11	42.38	–1.04	0.300
**Intransitive condition**	**–103.59**	**38.16**	**–2.72**	**0.007**
**Group**	**131.78**	**50.51**	**2.61**	**0.011**
Group × Plausible transitive condition	36.87	49.18	0.75	0.455
**Group × Intransitive condition**	**117.91**	**44.27**	**2.66**	**0.008**
*• Second pass reading time*				
*Intercept* (Baseline: L1group)	586.12	83.95		
Plausible transitive condition	–98.86	79.27	–1.25	0.213
Intransitive condition	–113.67	74.56	–1.53	0.128
Group	153.65	106.79	1.44	0.153
Group × Plausible transitive condition	22.27	90.18	0.25	0.805
Group × Intransitive condition	26.10	86.10	0.30	0.762
**Region 3 (*that*)**				
*• First pass reading time*				
*Intercept* (Baseline: L1group)	154.20	12.99		
Plausible transitive condition	8.60	16.08	0.54	0.593
Intransitive condition	4.99	16.94	0.30	0.768
**Group**	**103.01**	**15.68**	**6.57**	**<0.001**
Group × Plausible transitive condition	0.60	18.13	0.03	0.973
Group × Intransitive condition	–2.40	18.97	–0.13	0.899
*• Right–bounded reading time*				
*Intercept* (Baseline: L1group)	191.14	27.14		
Plausible transitive condition	20.31	20.92	0.97	0.332
Intransitive condition	15.16	22.41	0.68	0.499
**Group**	**102.50**	**20.27**	**5.06**	**<0.001**
Group × Plausible transitive condition	–23.37	23.63	–0.99	0.323
Group × Intransitive condition	–19.74	25.05	–0.79	0.431
*• Second pass reading time*				
*Intercept* (Baseline: L1group)	198.80	34.68		
Plausible transitive condition	–47.11	41.23	–1.14	0.254
Intransitive condition	21.66	42.91	0.51	0.614
Group	34.98	42.07	0.83	0.407
Group × Plausible transitive condition	48.37	45.94	1.05	0.293
Group × Intransitive condition	–40.84	47.46	–0.86	0.390
**Region 4 (*the writer*)**				
*• First pass reading time*				
*Intercept* (Baseline: L1group)	221.42	25.92		
Plausible transitive condition	–2.47	19.93	–0.12	0.901
Intransitive condition	2.60	19.78	0.13	0.900
**Group**	**307.34**	**29.75**	**10.33**	**<0.001**
Group × Plausible transitive condition	–10.71	26.51	–0.40	0.686
Group × Intransitive condition	–2.14	26.52	–0.08	0.936
*• Right–bounded reading time*				
*Intercept* (Baseline: L1group)	372.98	36.01		
Plausible transitive condition	3.86	22.60	0.17	0.864
Intransitive condition	27.47	22.52	1.22	0.223
**Group**	**251.11**	**40.15**	**6.26**	**<0.001**
Group × Plausible transitive condition	–18.00	30.00	–0.60	0.548
Group × Intransitive condition	–39.11	30.01	–1.30	0.193
*• Second pass reading time*				
*Intercept* (Baseline: L1group)	420.38	58.84		
Plausible transitive condition	–0.89	37.28	–0.02	0.981
Intransitive condition	–46.78	36.93	–1.27	0.206
Group	107.93	77.97	1.38	0.170
Group × Plausible transitive condition	15.01	49.67	0.30	0.762
Group × Intransitive condition	41.15	29.23	0.84	0.403
**Region 5 (*interviewed/smiled*)**				
*• First pass reading time*				
*Intercept* (Baseline: L1group)	216.11	16.38		
Plausible transitive condition	–2.96	12.56	–0.24	0.814
Intransitive condition	–9.74	12.48	–0.78	0.436
**Group**	**195.71**	**22.56**	**8.68**	**<0.001**
Group × Plausible transitive condition	1.72	17.04	0.10	0.920
**Group × Intransitive condition**	**42.48**	**17.07**	**2.49**	**0.013**
*• Right–bounded reading time*				
*Intercept* (Baseline: L1group)	269.69	18.56		
Plausible transitive condition	–10.81	13.98	–0.77	0.440
Intransitive condition	–20.27	13.93	–1.46	0.146
**Group**	**186.91**	**25.05**	**7.46**	**<0.001**
Group × Plausible transitive condition	–8.86	18.98	–0.47	0.641
**Group × Intransitive condition**	**43.52**	**19.03**	**2.29**	**0.022**
*• Second pass reading time*				
*Intercept* (Baseline: L1group)	236.31	48.20		
Plausible transitive condition	40.03	34.00	1.18	0.239
Intransitive condition	–4.87	34.30	–0.14	0.887
**Group**	**268.71**	**63.76**	**4.21**	**<0.001**
Group × Plausible transitive condition	–69.26	45.55	–1.52	0.129
Group × Intransitive condition	–51.03	45.95	–1.11	0.267
**Region 6 (*about*)**				
*• First pass reading time*				
*Intercept* (Baseline: L1group)	207.56	10.84		
Plausible transitive condition	4.98	10.20	0.49	0.625
Intransitive condition	–8.02	11.19	–0.72	0.474
**Group**	**67.25**	**14.21**	**4.73**	**<0.001**
**Group × Plausible transitive condition**	**–22.86**	**13.00**	**–1.76**	**0.079**
Group × Intransitive condition	–2.79	14.00	–0.20	0.842
*• Right–bounded reading time*				
*Intercept* (Baseline: L1group)	242.84	12.08		
Plausible transitive condition	–10.06	11.85	–0.85	0.400
**Intransitive condition**	–**41.16**	**13.11**	–**3.14**	**0.002**
**Group**	**56.36**	**15.88**	**3.55**	**<0.001**
Group × Plausible transitive condition	–7.35	15.13	–0.49	0.627
Group × Intransitive condition	23.55	16.42	1.43	0.152
*• Second pass reading time*				
*Intercept* (Baseline: L1group)	220.74	37.21		
Plausible transitive condition	21.16	34.79	0.61	0.545
**Intransitive condition**	**–75.11**	**35.92**	**–2.09**	**0.038**
**Group**	**118.06**	**48.67**	**2.43**	**0.018**
Group × Plausible transitive condition	–30.22	44.63	–0.68	0.500
Group × Intransitive condition	–36.61	44.77	–0.82	0.415
**Region 7 (*at the conference*)**				
*• First pass reading time*				
*Intercept* (Baseline: L1group)	399.85	38.69		
Plausible transitive condition	–11.89	25.37	–0.47	0.640
Intransitive condition	35.10	25.28	1.39	0.165
**Group**	**324.39**	**50.01**	**6.49**	**<0.001**
Group × Plausible transitive condition	–41.18	35.85	–1.15	0.251
Group × Intransitive condition	–12.15	35.63	–0.34	0.733
*• Right–bounded reading time*				
*Intercept* (Baseline: L1group)	822.01	95.28		
**Plausible transitive condition**	**104.85**	**42.79**	**2.45**	**0.014**
Intransitive condition	39.55	42.39	0.93	0.351
**Group**	**538.48**	**69.30**	**4.47**	**<0.001**
**Group × Plausible transitive condition**	–**152.13**	**60.52**	–**2.51**	**0.012**
Group × Intransitive condition	–34.69	59.95	–0.58	0.563

*The results with p-values less than 0.05 are shown in bold.*

#### Region 2 (The Celebrity/The Letter)

Right-bounded times in this region showed an interaction between Group and Intransitive condition. The interaction indicates that there was a difference between the two groups in the way that the sentences with the intransitive verb were processed compared to the processing of the baseline Impossible transitive condition. The simple effect analysis showed that an effect of Intransitive condition was observed only with the L1 group but not with the L2 group (*p* = 0.523). With the L1 group, the reading time was shorter in the Intransitive condition than in the Impossible transitive condition (Intransitive: 394 ms, Impossible transitive: 474 ms). Since the lexical information is consistent between the Impossible transitive condition and the Intransitive condition up to Region 4 (e.g., … *the letter that the writer*…), the effect of Intransitive condition in this region with the L1 group is most likely a parafoveal effect. When the upcoming verb was intransitive, the L1 group processed the sentence faster and continued on to the following region quickly compared to when the upcoming verb was optionally transitive. No such parafoveal effect of intransitive information was found with the L2 group.

First pass and right-bounded times in this region also showed a main effect of Group, showing that the reading times of this region in these measures were longer with the L2 group than the L1 group.

#### Region 3 and 4 (That the Writer)

A main effect of Group was found in first pass times and right-bounded times in Region 3 and 4. These results indicate that the L2 group was overall slower to process information in these regions compared to the L1 group.

#### Region 5 (Interviewed/Smiled)

**FIGURE 1:**
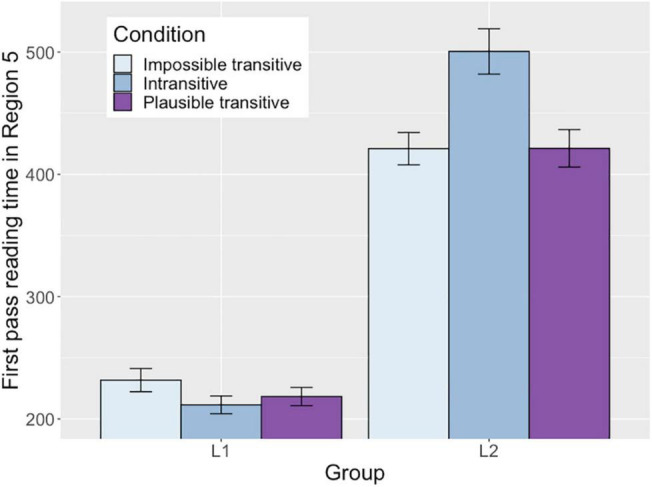
First pass reading time in Region 5 in each condition in each group.

First pass and right-bounded times in this region showed an interaction between Group and Intransitive condition. The interaction indicates that there was a difference between the two groups in the way that the sentences with the intransitive verb were processed compared to the processing of the baseline Impossible transitive condition. The simple effect analysis indicated an effect of Intransitive condition with the L2 group (β = 32.75, *SE* = 11.74, *t* = 2.79, *p* = 0.005) but there was no effect of Intransitive condition with the L1 group. This demonstrates that the reading time for the intransitive verbs was significantly longer than that for the optionally transitive verbs in the Impossible transitive condition only with the L2 group (Intransitive: 500 ms, Impossible transitive: 421 ms for L2, Intransitive: 212 ms, Impossible transitive: 232 ms for L1). Similarly, the interaction between Group and Intransitive condition in right-bounded reading time also showed a marginal simple effect of Intransitive condition with the L2 group (β = 23.25, *SE* = 13.15, *t* = 1.77, *p* = 0.077) but there was no effect of Intransitive condition with the L1 group. This demonstrates that the L2 group spent longer time to process the verb before they proceeded to the following regions in the Intransitive verb condition than in the Impossible transitive verb condition, but this effect was not observed with the L1 group (Intransitive: 550 ms, Impossible transitive: 461 ms for L2, Intransitive: 260 ms, Impossible transitive: 284 ms for L1). Figure 1 illustrates the different reading patterns in first pass times between the two groups. As shown in the figure, the L2 group showed increased reading time for the Intransitive condition compared to other two conditions.

The longer reading time for the intransitive verbs in first pass and right-bounded times with the L2 group indicates that L2 learners experienced processing difficulty on encountering an intransitive verb compared to when they saw a transitive verb. The L2 group’s results that they required longer reading time to process intransitive verbs most likely reflect that L2 learners had a strong expectation for an upcoming verb to be a transitive verb that can take the preceded NP (*the celebrity*/*the letter*) as a direct object. As a result, they experienced processing difficulty on encountering an intransitive verb that did not match with the expectations they had made. Importantly, the different processing patterns between the Intransitive condition and the Impossible transitive condition provide evidence for the use of verb’s subcategorization information in L2 processing. If the increased reading times in the Intransitive condition were due to the semantically anomalous interpretation of the direct object analysis (e.g., *smiled the letter*), then the reading times in the Impossible transitive condition would also be similar to that in the Intransitive condition because the direct object analysis in this condition also causes semantically anomalous interpretation (e.g., *interviewed the letter*). Thus, the difference between the two conditions indicate that the L2 group distinguished obligatory intransitive verbs from optionally transitive verbs in online structure analysis. As was confirmed by the results of the simple effect of Intransitive condition, processing difficulty on encountering the intransitive verbs was observed only with the L2 group but not with the L1 group.

In addition, there was a main effect of Group in all measures, showing that the overall reading times with L2 group were longer than the L1 group in this region.

#### Region 6 (About)

Right-bounded times in this region showed a main effect of Intransitive condition, demonstrating that the time spent at this region, including re-reading of the earlier regions before participants proceeded to the following region was shorter in the Intransitive condition than in the Impossible transitive condition regardless of the group (Intransitive: 538 ms, Impossible transitive: 578 ms). The same effect of Intransitive condition was also observed in second pass times, showing that re-reading time in this region was shorter in the Intransitive condition compared to the Impossible transitive condition (Intransitive: 525 ms, Impossible transitive: 494 ms). These results most likely reflect a smaller cost for structural revision in the Intransitive condition as the verb’s intransitivity information was helpful for both groups in reaching the correct analysis without structural reanalysis.

In addition, first pass reading time in this region showed a marginal interaction between Group and Plausible transitive condition. Although the interaction did not reach the level of significance, the main effect of Group in the first pass times shows that the L2 group’s reading time was significantly longer compared to the L1 group’s. This suggests the possibility that the marginally significant interaction between Group and Plausible transitive condition might indicate different reading patterns between the two groups. We explored this possibility by conducting a simple effect analysis with the L2 group. The results showed there was a marginal effect of Plausible transitive condition with the L2 group (β = –17.41, *SE* = 9.40, *t* = –1.85, *p* = 0.064), while there was no effect of Plausible transitive condition with the L1 group. The results demonstrates that the L2 group tended to spend longer time reading the post-verbal region in the Impossible transitive condition compared to the Plausible transitive condition, and this tendency was observed only with the L2 group (Impossible transitive: 292 ms, Plausible transitive: 275 ms for L2, Impossible transitive: 233 ms, Plausible transitive: 233 ms for L1). This most likely reflects that the L2 group showed a spill-over effect in this region when the verb information in the previous region resulted in semantically anomalous direct object interpretation in the Impossible transitive condition (e.g., *interviewed the letter*). The fact that the interaction failed to reach full significance might be, at least partly, due to low statistical power in our analysis. Our study could be considered as two sets of separate studies (L1 and L2 groups), with each having 29 and 31 participants. Although the number of participants is not small relative to other L2 studies, it may be possible that the sample size was not large enough for reliably observing an interaction in the unified analysis with a within-participants sentence condition (Impossible transitive vs. Plausible transitive) and a between-participants group condition (L1 and L2). At the same time, low statistical power could also increase the risk of Type I error so that we should be careful not to overinterpret this finding.

As in the other regions, a main effect of Group was observed in all reading measures in this region, again showing that the overall reading times with L2 group were longer than the L1 group.

#### Region 7 (At the Conference)

**FIGURE 2:**
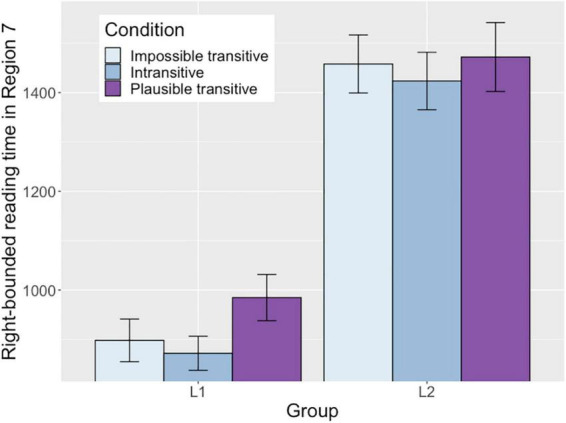
Right-bounded reading time in Region 7 in each condition in each group.

Right-bounded reading time in this region showed an effect of Plausible transitive condition as well as an interaction between Group and Plausible transitive condition. From the main effect of Plausible transitive condition, it was demonstrated that the L1 group’s reading times in this region were longer in the Plausible transitive condition than in the Impossible transitive condition. The simple effect analysis with the L2 group showed this effect was not significant with the L2 group (*p* = 0.269). The longer right-bounded times in this region with the L1 group most likely reflect that the L1 group had adopted the direct object analysis in the Plausible transitive condition up to this region, and regressed to the earlier regions on encountering information that was not consistent with the analysis. This is because the direct object analysis in the Plausible transitive condition is possible up to the previous post-verbal region (e.g., *The writer interviewed the celebrity about*…[*her next concert*]), but this analysis becomes impossible in the current sentence-final region (e.g., *The writer interviewed the celebrity about at the conference). Figure 2 illustrates the different reading patterns in right-bounded times between the two groups. As shown in the figure, only the L1 group showed increased reading time for the Plausible transitive condition compared to the Impossible transitive condition.

The first pass and right-bounded times in this region showed a main effect of Group, reflecting that these reading times were longer with the L2 group compared to the L1 group.

### Summary of the Results

*The results revealed both similarities and differences between the L1 group and the L2 group in the use of subcategorization information during the processing of the filler-gap dependency structure.*

First, the L2 group was surprised to encounter the intransitive verbs. The first pass and the right-bounded reading times at the verb region (Region 5) showed that only the L2 group showed longer reading times for the intransitive verbs compared to the transitive verbs. This demonstrates that the L2 group required extra time to process an intransitive verb, most likely reflecting their strong expectations for the upcoming verb to be a transitive verb that takes a preceded NP as the verb’s direct object. Importantly, the results cannot be explained by the possibility that the L2 group adopted an direct object in the Intransitive condition by ignoring the verb’s subcategorization information and experienced processing difficulty due to the semantically anomalous interpretation. If this were the case, the Impossible transitive condition should also show increased reading times for the same reason at the verb region. Thus, the difference between the Intransitive condition and the Impossible transitive condition provides evidence for the use of verb’s subcategorization information in L2 processing. With the L1 group, a very early effect of Intransitive condition was observed as a parafoveal effect in Region 2.

Second, there was a suggestion for a semantic anomaly effect with the L2 group at the spill-over region. The first pass times at the post-verbal region (Region 6) showed that only the L2 group experienced processing cost in the Impossible transitive condition compared to the Plausible transitive condition. Although the effect was marginal, the L2 group showed a numerically longer reading time when the verb information in the previous verb region resulted in a semantically anomalous direct object analysis interpretation as in *the writer interviewed the letter* compared to when the direct object analysis was semantically plausible as in *the writer interviewed the celebrity*. The cost for semantic implausibility was not observed with the L1 group.

Third, both groups processed sentences differently depending on the verb type. At the post-verbal region (Region 6), right-bounded and second pass times showed that both L1 group and L2 group spent longer to read this region in the Plausible transitive condition than the Intransitive condition. This indicates that both groups experienced a larger reanalysis cost for the Plausible transitive condition, and that neither group was forced to perform structural revision in the Intransitive condition. The results provide evidence that both L1 and L2 groups used the subcategorization frame information of the verbs in structural analysis.

Finally, the L1 group showed processing difficulty in revising the initial direct object analysis in the Plausible transitive condition. The right-bounded times in the sentence-final region (Region 7) showed that L1 group regressed to the earlier regions when the direct object analysis became infeasible in the Plausible transitive condition. The L1 group initially analyzed the preceding NP as the verb’s direct object in the Plausible transitive condition (e.g., *The writer interviewed the celebrity* …), but on encountering the sentence-final prepositional phrase (e.g., *at the conference*), they noticed that the analysis they had adopted was inconsistent with the sentence continuation. They thus revised the preposition as a part of a prepositional phrase (e.g., *smiled about* as in *the writer smiled about the letter*…).

## General Discussion

The current study addressed the question of whether L2 learners are able to exploit the verb’s subcategorization information in online syntactic processing. We contrasted three possible accounts; the initial inaccessibility account, the intransitivity overriding account, and the fuzzy subcategorization frame account. The initial inaccessibility account that L2 learners possess the correct subcategorization knowledge, but intransitivity information cannot be immediately accessed during online structural analysis. The intransitivity overriding account that L2 learners can access subcategorization information but the use of intransitivity information is overridden by the presence of a postverbal noun due to the strong preference for analyzing the noun as a verb’s direct object. Under this account, it is predicted that L2 learners would be able to use intransitivity information as long as an NP does not directly appear following an intransitive verb. The fuzzy subcategorization frame account that L2 learners’ structural information is represented somewhat in a fuzzy way so that it allows certain ambiguities in the prescription of subcategorization frames. Under this account, L2 learners are expected to be unable to make structural judgment based on verb’s subcategorization information. As a result, L2 learners would tolerate the incorrect transitive use for an obligatory intransitive verb and form a plausible interpretation, causing little or no processing difficulty. We tested these accounts by examining the processing of the locally ambiguous unbounded dependency structure with native speakers and Japanese-English L2 learners. The results from the eye-tracking experiment indicated that the filler-gap dependency structure was processed using verb’s subcategorization information by both native speakers and L2 learners. The pattern of the results, however, was not identical between the two groups and we now discuss both the common findings and the differences including the implications of these results.

The results indicated that both groups processed sentences differently depending on the verb’s subcategorization information. Both groups experienced reduced processing cost when the verb was intransitive as indexed by shorter reading times at the post-verbal region in the Intransitive condition than the Transitive conditions. This reflects that verb’s intransitivity information helped readers to adopt the correct sentence structure; they adopted the direct object analysis only when the verb was optionally transitive but not when the verb was obligatory intransitive. It was also shown that L2 learners experienced an extra cost in processing intransitive verbs compared to optionally transitive verbs. This was indexed by a longer reading time at the verb region in the intransitive condition than in the transitive conditions. This reflects that L2 learners had a strong expectation for the upcoming verb to be transitive, and experienced difficulty on encountering an intransitive verb. These results provide evidence for the use of verb’s subcategorization information in L2 processing, and are incompatible with the previous studies which found that intransitivity information was ignored during L2 comprehension. This is most likely because previous studies used a structure in which an NP appears directly following a verb, and this structure perhaps triggered an L2 learner’s strong preference for a direct object analysis, which overrode intransitive subcategorization restrictions in L2 processing. The results of the current study thus provide support for the overriding account, showing that L2 learners can immediately use intransitivity information to guide their structural analysis when there are no overriding constraints. One possible drawback is that different verbs had to be used for the transitive and intransitive conditions, and any potential differences in the frequency or the number of characters between the two types of the verbs could have contributed to the difference in reading times between the conditions. This is perhaps most relevant for the finding of longer reading times at the verb region with the intransitive verbs than with the transitive verbs with L2 learners. We checked the familiarity of the two sets of the verbs using a L2 database (Yokokawa, 2006) and confirmed that there was no reliable difference between the transitive and intransitive verbs. This, however, is a null effect based on one database and we thus cannot reject the possibility for the influence of lexical factors completely.

The results also revealed that L2 learners experienced immediate processing disruption on encountering an intransitive verb. This processing pattern was unique to the L2 learners, and we interpret this finding as being consistent with the hyper-active gap filling hypothesis proposed by Omaki et al. (2015). Although the hypothesis was originally proposed for L1 processing, we think it is plausible that L2 learners show a stronger tendency for the hyper-active gap filling because L2 learners would rely more on category-general transitive knowledge in processing sentences in their L2 (see also Omaki and Schulz, 2011). As discussed in the mono-transitivity information as category-general hypothesis in the study of Van Gompel et al. (2012), transitive information applies to almost all verbs whereas the occurrence of an intransitive structure is much less frequent (Roland et al., 2007). Considering the limited exposure to L2, it is reasonable to think that L2 learners’ experience with intransitive verbs is even more limited compared to the native speakers’. Thus, the lexically specific knowledge of whether a particular verb should be used in an intransitive structure is less solid with L2 learners, and this leads them to have strong expectations for a more general transitive structure. As a result, L2 learners were surprised to see an intransitive verb because it violates the expectation they generated prior to the verb.

The reading patterns at the post-verbal region also suggested that L2 learners experienced processing difficulty due to semantically anomalous direct object analysis in the Impossible transitive condition at the spill-over region with L2 learners. Native speakers did not show processing difficulty in the Impossible transitive condition, most likely reflecting that native speakers adopted the correct analysis using semantic information without processing cost. The finding that the cost for semantic mismatch was observed only with L2 learners but not with native speakers suggest that native speakers immediately revised the structure for the correct structure when the direct object analysis was semantically anomalous. In contrast, L2 learners adopted the direct object analysis even when the interpretation was semantically anomalous and experienced processing difficulty. The results are consistent with the previous research, which showed that L2 learners stick to the initially adopted semantically anomalous direct object analysis and this causes a delay in revising the sentence to the correct structure in L2 processing (Roberts and Felser, 2011).

With native speakers, processing cost due to structural reanalysis was observed at the sentence final region in the Plausible transitive condition. The direct object analysis in the Plausible transitive condition was plausible up to this region, and when native speakers encountered information that was inconsistent with the analysis in the sentence-final region, they spent longer time re-reading the earlier regions. With L2 learners, no evidence for structural reanalysis cost in the Plausible transitive condition was observed. This might suggest the possibility that L2 learners did not reach the correct structural interpretation in the Plausible transitive condition. Some previous studies have suggested that readers do not always engage in fully detailed analysis but instead use heuristics in processing sentences. They have shown that readers often preserve an initially adopted incorrect analysis even after the initial analysis turns out to be incorrect (Christianson et al., 2001; Van Gompel et al., 2006). There is also a finding that readers perform incomplete reanalysis more with complex sentence structure when the semantic information supports the initial incorrect analysis (Nakamura and Arai, 2015). The view that language users do not always build a complete sentence representation, known as the Good Enough approach, suggests the possibility that our participants in the present study did not ultimately reach the correct analysis. Given that there was no evidence for structural reanalysis cost in the Plausible transitive condition with L2 learners, L2 learner’s structural reanalysis in the Plausible transitive condition may possibly have ended up incomplete, with the incorrect direct object analysis retained as the final interpretation of the sentences. In the experiment, we did not include questions about the final interpretation because they would draw participants’ attention to the structural ambiguity (e.g., *Did the writer interviewed the celebrity?* for 7a), causing them to notice the purpose of our experiment. We therefore cannot know what final representation they constructed with these sentences and this issue is left to future research.

The initial inaccessibility account predicted that L2 learners cannot use verb’s subcategorization information during online sentence processing even though they have the experience-based knowledge about possible argument structures. This account was dismissed because our results provided clear evidence that intransitive verbs and transitive verbs were processed differently by L2 learners. The fuzzy subcategorization frame representation account predicted that L2 learners’ fuzzy representation of argument structure information would cause them not to exclude the argument structure that is not a part of the verb’s subcategorization frame. As a result, L2 learner’s tolerate the incorrect transitive use for an intransitive structure, and form an interpretation out of the ungrammatical sentence using semantic information. This account was also dismissed because our results showed that L2 learners experienced a facilitatory effect due to the semantically anomalous direct object analysis only in the Impossible transitive condition but not in the Intransitive condition. Instead, our results provided support for the intransitivity overriding account, which predicted that L2 learners are able to apply the knowledge as long as there is no overriding information, i.e., when the sentence structure does not have an NP directly following an intransitive verb.

We now explore the possibility of an alternative explanation that the use of verb subcategorization information by our L2 learners may be accounted for by L1 transfer. In Japanese, sentence structure is typically expressed by two grammatical features; case marking and verb inflection (Jacobsen, 1991). For example, to use the verb *break* (*kowasu*) is transitive in Japanese; the verb should be preceded by an NP with the accusative case particle *o* as in *kabin o kowasu* (vase-ACC break, “break a vase”). For the same verb to be used intransitively, the verb should occur with the suffix -*eru* as in *kabin ga kowareru* (vase-NOM break, “the vase broke”) in which case the verb is preceded by the NP with the nominative case particle *ga*. However, the suffixes used to mark transitive and intransitive forms of a verb are not always consistent across all verbs and it is thus not always possible to tell the structure from the verb form (For example, the suffix -*eru* is used to mark an intransitive form for the verb *kowasu* “break” as in the example above, but the same suffix *-eru* is used to mark a transitive counterpart for the verb *aku* “open,” as in *akeru*). Furthermore, Japanese allows arguments to be expressed implicitly as in *Kare-ga kowashita, “*He broke (something).” Thus, only when the NP with the accusative case marker is explicitly present in the sentence, can one rule out the intransitive structure. In the filler-gap dependency structure used in the current study, an accusative NP preceded the verb, which is in a way similar to the Japanese head-final construction. Thus, it may be possible that our Japanese participants saw some similarity between the cleft sentences used in the current study and Japanese transitive sentences even though the NP in the former does not contain case marker, which may have contributed to some extent to the prediction about the upcoming transitive verb. The degree of contribution of L1 transfer on our findings needs to be confirmed in future research by testing different populations of L2 learners.

In sum, the results of the current study together demonstrated that L2 learners are able to use lexically specific intransitivity information to guide their structural analyses when an NP was not present directly following a verb, thus providing support for the overriding account. The overriding account can also account for the previous studies that failed to observe an effect of subcategorization information in L2 processing; L2 learners possess subcategorization knowledge for individual verbs, but their intransitivity information often failed to guide the initial syntactic analysis. Our study, together with previous studies, suggested that the intransitivity information would be overridden when an NP appeared directly following the verb due to a strong bias toward the direct object analysis but can be used to process the sentence structure when the NP is dislocated from the post-verbal position and preceded the verb as in a filler-gap dependency structure.

## Conclusion

This study investigated the effect of lexically specific verb information as well as semantic information in processing a temporarily ambiguous unbounded dependency structure with Japanese speakers learning English as an L2. Previous research that examined the use of the verb’s subcategorization information in L2 processing showed that L2 learners ignore intransitivity information in online structural analyses. The current study examined the possibility that verbal intransitivity information is in fact accessed in L2 parsing but the information tends to get overridden by a strong preference to analyze an NP directly following a verb as the verb’s direct object. The results of eye-tracking experiments in reading unbounded dependency structures showed that L2 learners treated intransitive verbs differently from transitive verbs in incremental structural analysis. It was also revealed that L2 learners had stronger expectations for a transitive structure than native speakers, and that L2 learners required a longer time to revise the sentence structure when the semantic information did not support the initially adopted analysis. To conclude, this study provided evidence for the use of verb subcategorization information as well as semantic information in L2 processing, demonstrating that verb’s subcategorization information is not ignored in L2 processing and L2 learners can make use of verb information in situations where no overriding information is present.

## Data Availability Statement

The raw data supporting the conclusions of this article will be made available by the authors, without undue reservation.

## Ethics Statement

The studies involving human participants were reviewed and approved by the Massachusetts Institute of Technology Committee on the Use of Humans as Experimental Subjects. The patients/participants provided their written informed consent to participate in this study.

## Author Contributions

CN, MA, YH, and SF contributed to the conception and design of the study. CN organized the data collection and performed the statistical analysis. MA supported the statistical analysis process. YH and SF contributed to interpreting the results from the statistical analysis. CN and MA wrote the first draft of the manuscript. All authors contributed to manuscript revision, read, and approved the submitted version.

## Conflict of Interest

The authors declare that the research was conducted in the absence of any commercial or financial relationships that could be construed as a potential conflict of interest.

## Publisher’s Note

All claims expressed in this article are solely those of the authors and do not necessarily represent those of their affiliated organizations, or those of the publisher, the editors and the reviewers. Any product that may be evaluated in this article, or claim that may be made by its manufacturer, is not guaranteed or endorsed by the publisher.

## Footnotes

1 For the verbs used in Region 5, we conducted another analysis to see whether L2 learners’ familiarity with the verbs was different between the two types of the verbs (transitive and intransitive). For this analysis, we used a database in which familiarity for 3,000 English words is rated by Japanese learners of English (Yokokawa, 2006). The average familiarity rating for the verbs used in the transitive conditions was 5.67 in the 7 point scale (*SD* = 0.66), and that for the verbs used in the intransitive condition was 5.87 (*SD* = 0.67). The result of an unpaired *t*-test showed that there was no difference in L2 learners’ familiarity with the verbs used in the transitive and intransitive conditions (*p* = 0.161).
