# Supplementary material for: L2 Learners Do Not Ignore Verb’s Subcategorization Information in Real-Time Syntactic Processing
Source: Front Psychol. 2022 Jan 20;12:689137. doi: 10.3389/fpsyg.2021.689137 (PMC8810650; doi:10.3389/fpsyg.2021.689137)
Supplement: Supplementary file 1 [file Data_Sheet_1.docx]

Supplementary Table 1. Target items used in the experiment.

| Item | Condition | Sentence | First NP frequency | Verb frequency |
| --- | --- | --- | --- | --- |
| 1 | Plausible transitive | That's the class that the lecturer taught about during the semester. | 17694 | 2721 |
| 1 | Impossible transitive | That's the building that the lecturer taught about during the semester. | 15842 | 2721 |
| 1 | Intransitive | That's the building that the lecturer complained about during the semester. | 15842 | 1299 |
| 2 | Plausible transitive | That's the journal that the man wrote about in the library. | 2333 | 10497 |
| 2 | Impossible transitive | That's the girl that the man wrote about in the library. | 14383 | 10497 |
| 2 | Intransitive | That's the girl that the man screamed at in the library. | 14383 | 425 |
| 3 | Plausible transitive | That's the story that the mother told about at the party. | 13061 | 28590 |
| 3 | Impossible transitive | That's the food that the mother told about at the party. | 18437 | 28590 |
| 3 | Intransitive | That's the food that the mother laughed about at the party. | 18437 | 1787 |
| 4 | Plausible transitive | That's the boy that the woman consulted about in the evening. | 12560 | 1166 |
| 4 | Impossible transitive | That's the house that the woman consulted about in the evening. | 47950 | 1166 |
| 4 | Intransitive | That's the house that the woman screamed at in the evening. | 47950 | 425 |
| 5 | Plausible transitive | That's the magazine that the housewife read about in the garden. | 4450 | 16575 |
| 5 | Impossible transitive | That's the businessman that the housewife read about in the garden. | 930 | 16575 |
| 5 | Intransitive | That's the businessman that the housewife smiled at in the garden. | 930 | 928 |
| 6 | Plausible transitive | That's the youngster that the manager instructed about before the event. | 483 | 342 |
| 6 | Impossible transitive | That's the door that the manager instructed about before the event. | 23068 | 342 |
| 6 | Intransitive | That's the door that the manager shouted at before the event. | 23068 | 899 |
| 7 | Plausible transitive | That's the blanket that the girl hid inside during the night. | 1065 | 2085 |
| 7 | Impossible transitive | That's the forest that the girl hid inside during the night. | 7084 | 2085 |
| 7 | Intransitive | That's the forest that the girl walked in during the night. | 7084 | 6147 |
| 8 | Plausible transitive | That's the noise that the neighbor heard about in the street. | 4351 | 12787 |
| 8 | Impossible transitive | That's the picture that the neighbor heard about in the street. | 10083 | 12787 |
| 8 | Intransitive | That's the picture that the neighbor laughed at in the street. | 10083 | 1787 |
| 9 | Plausible transitive | That's the question that the interviewer asked about at the exhibition. | 14092 | 18485 |
| 9 | Impossible transitive | That's the painting that the interviewer asked about at the exhibition. | 3757 | 18485 |
| 9 | Intransitive | That's the painting that the interviewer cried about at the exhibition. | 3757 | 1468 |
| 10 | Plausible transitive | That's the artist that the designer visited with during the vacation. | 3921 | 4794 |
| 10 | Impossible transitive | That's the luggage that the designer visited with during the vacation. | 619 | 4794 |
| 10 | Intransitive | That's the luggage that the designer walked with during the vacation. | 619 | 6147 |
| 11 | Plausible transitive | That's the celebrity that the writer interviewed about at the conference. | 401 | 439 |
| 11 | Impossible transitive | That's the letter that the writer interviewed about at the conference. | 12993 | 439 |
| 11 | Intransitive | That's the letter that the writer smiled about at the conference. | 12993 | 928 |
| 12 | Plausible transitive | That's the politician that the reporter informed about at the meeting. | 1034 | 1453 |
| 12 | Impossible transitive | That's the car that the reporter informed about at the meeting. | 26343 | 1453 |
| 12 | Intransitive | That's the car that the reporter complained about at the meeting. | 26343 | 1299 |
| 13 | Plausible transitive | That's the course that the professor lectured about in the classroom. | 19181 | 152 |
| 13 | Impossible transitive | That's the radio that the professor lectured about in the classroom. | 8443 | 152 |
| 13 | Intransitive | That's the radio that the professor listened to in the classroom. | 8443 | 5356 |
| 14 | Plausible transitive | That's the patient that the doctor advised about in the hallway. | 6600 | 2001 |
| 14 | Impossible transitive | That's the pacemaker that the doctor advised about in the hallway. | 54 | 2001 |
| 14 | Intransitive | That's the pacemaker that the doctor walked with in the hallway. | 54 | 6147 |
| 15 | Plausible transitive | That's the child that the teacher dressed for in the weekend. | 23486 | 916 |
| 15 | Impossible transitive | That's the party that the teacher dressed for in the weekend. | 38378 | 916 |
| 15 | Intransitive | That's the party that the teacher agreed about in the weekend. | 38378 | 7935 |
| 16 | Plausible transitive | That's the teammate that the wrestler attacked with during the match. | 27 | 1697 |
| 16 | Impossible transitive | That's the rope that the wrestler attacked with during the match. | 1490 | 1697 |
| 16 | Intransitive | That's the rope that the wrestler jumped over during the match. | 1490 | 1482 |
| 17 | Plausible transitive | That's the captain that the soldier killed for during the war. | 5165 | 4239 |
| 17 | Impossible transitive | That's the order that the soldier killed for during the war. | 19441 | 4239 |
| 17 | Intransitive | That's the order that the soldier disagreed about during the war. | 19441 | 724 |
| 18 | Plausible transitive | That's the golfer that the instructor assisted with at the club. | 363 | 2468 |
| 18 | Impossible transitive | That's the computer that the instructor assisted with at the club. | 13446 | 2468 |
| 18 | Intransitive | That's the computer that the instructor disagreed about at the club. | 13446 | 724 |
| 19 | Plausible transitive | That's the volunteer that the friend supported with for the funding. | 632 | 9578 |
| 19 | Impossible transitive | That's the money that the friend supported with for the funding. | 36031 | 9578 |
| 19 | Intransitive | That's the money that the friend agreed about for the funding. | 36031 | 7935 |
| 20 | Plausible transitive | That's the letter that the couple wrote about at the wedding. | 12993 | 10497 |
| 20 | Impossible transitive | That's the flower that the couple wrote about at the wedding. | 2108 | 10497 |
| 20 | Intransitive | That's the flower that the couple argued about at the wedding. | 2108 | 4170 |
| 21 | Plausible transitive | That's the secret that the woman learned about at the school. | 2048 | 8042 |
| 21 | Impossible transitive | That's the garden that the woman learned about at the school. | 10718 | 8042 |
| 21 | Intransitive | That's the garden that the woman argued about at the school. | 10718 | 4170 |
| 22 | Plausible transitive | That's the cellphone that the artist answered about before the concert. | 9 | 4901 |
| 22 | Impossible transitive | That's the music that the artist answered about before the concert. | 14433 | 4901 |
| 22 | Intransitive | That's the music that the artist listened to before the concert. | 14433 | 5356 |
| 23 | Plausible transitive | That's the e-mail that the secretary replied about after the meeting. | 172 | 1162 |
| 23 | Impossible transitive | That's the bathroom that the receptionist replied about after the meeting. | 2308 | 1162 |
| 23 | Intransitive | That's the bathroom that the receptionist cried in after the meeting. | 2308 | 1468 |
| 24 | Plausible transitive | That's the player that the athlete called about after the race. | 5401 | 12522 |
| 24 | Impossible transitive | That's the fence that the athlete called about after the race. | 1613 | 12522 |
| 24 | Intransitive | That's the fence that the athlete jumped over after the race. | 1613 | 1482 |

Supplementary Table 2. Mean reading time (and standard errors) in milliseconds for first pass, right-bounded, and second pass measures in Region 2 to Region 7.

|  | *First* pass | *Right-bounded* | *Second pass* |
| --- | --- | --- | --- |
| **Region 2 (*the celebrity/the letter*)** |  |  |  |
| **L1 group** |  |  |  |
| Plausible transitive | 215 (25) | 480 (26) | 556 (46) |
| Impossible transitive | 211 (16) | 474 (36) | 550 (63) |
| Intransitive | 210 (18) | 394 (41) | 456 (104) |
| **L2 group** |  |  |  |
| Plausible transitive | 647 (42) | 726 (59) | 793 (96) |
| Impossible transitive | 562 (28) | 625 (47) | 795 (96) |
| Intransitive | 542 (28) | 612 (31) | 708 (78) |
| **Region 3 (*that*)** |  |  |  |
| **L1 group** |  |  |  |
| Plausible transitive | 175 (11) | 215 (18) | 152 (42) |
| Impossible transitive | 164 (19) | 209 (24) | 194 (33) |
| Intransitive | 188 (18) | 250 (32) | 208 (38) |
| **L2 group** |  |  |  |
| Plausible transitive | 278 (11) | 316 (15) | 273 (29) |
| Impossible transitive | 272 (10) | 317 (15) | 306 (39) |
| Intransitive | 282 (14) | 313 (16) | 261 (39) |
| **Region 4 (*the writer*)** |  |  |  |
| **L1 group** |  |  |  |
| Plausible transitive | 235 (14) | 389 (27) | 449 (48) |
| Impossible transitive | 247 (17) | 420 (31) | 460 (39) |
| Intransitive | 134 (11) | 414 (20) | 387 (34) |
| **L2 group** |  |  |  |
| Plausible transitive | 552 (32) | 649 (40) | 648 (82) |
| Impossible transitive | 564 (30) | 664 (37) | 647 (84) |
| Intransitive | 611 (38) | 683 (43) | 585 (75) |
| **Region 5 (*interviewed/smiled*)** |  |  |  |
| **L1 group** |  |  |  |
| Plausible transitive | 218 (18) | 270 (27) | 306 (60) |
| Impossible transitive | 232 (24) | 284 (29) | 256 (51) |
| Intransitive | 212 (19) | 260 (24) | 247 (57) |
| **L2 group** |  |  |  |
| Plausible transitive | 421 (20) | 468 (47) | 581 (78) |
| Impossible transitive | 421 (19) | 461 (43) | 557 (67) |
| Intransitive | 500 (29) | 550 (56) | 531 (96) |
| **Region 6 (*about*)** |  |  |  |
| **L1 group** |  |  |  |
| Plausible transitive | 233 (26) | 257 (27) | 300 (68) |
| Impossible transitive | 233 (26) | 262 (27) | 245 (59) |
| Intransitive | 221 (27) | 244 (33) | 175 (50) |
| **L2 group** |  |  |  |
| Plausible transitive | 275 (21) | 297 (25) | 376 (51) |
| Impossible transitive | 292 (22) | 316 (25) | 400 (46) |
| Intransitive | 273 (18) | 294 (22) | 290 (46) |
| **Region 7 (*at the conference*)** |  |  |  |
| **L1 group** |  |  |  |
| Plausible transitive | 430 (65) | 985 (132) | - |
| Impossible transitive | 440 (66) | 898 (119) | - |
| Intransitive | 463 (64) | 872 (97) | - |
| **L2 group** |  |  |  |
| Plausible transitive | 729 (83) | 1472 (193) | - |
| Impossible transitive | 753 (80) | 1458 (162) | - |
| Intransitive | 800 (84) | 1423 (161) | - |
